# Disease-modifying effect of intravenous immunoglobulin in an experimental model of epilepsy

**DOI:** 10.1038/srep40528

**Published:** 2017-01-11

**Authors:** Min Chen, Thiruma V. Arumugam, Gayeshika Leanage, Quang M. Tieng, Ashwin Yadav, Jeremy F. P. Ullmann, David T. She, Vy Truong, Marc J. Ruitenberg, David C. Reutens

**Affiliations:** 1 Centre for Advanced Imaging, The University of Queensland, Brisbane, 4072, Australia; 2 School of Biomedical Sciences, The University of Queensland, Brisbane, 4072, Australia; 3 Department of Physiology, Yong Loo Lin School of Medicine, The National University of Singapore, 117597, Singapore; 4 Queensland Brain Institute, The University of Queensland, Brisbane, 4072, Australia

## Abstract

Novel therapies that prevent or modify the development of epilepsy following an initiating brain insult could significantly reduce the burden of this disease. In light of evidence that immune mechanisms play an important role in generating and maintaining the epileptic condition, we evaluated the effect of a well-established immunomodulatory treatment, intravenous immunoglobulin (IVIg), on the development of epilepsy in an experimental model of epileptogenesis. In separate experiments, IVIg was administered either before (pre-treatment) or after (post-treatment) the onset of pilocarpine status epilepticus (SE). Our results show that both pre- and post-treatment with IVIg attenuated acute inflammation in the SE model. Specifically, IVIg reduced local activation of glial cells, complement system activation, and blood-brain barrier damage (BBB), which are all thought to play important roles in the development of epilepsy. Importantly, post-treatment with IVIg was also found to reduce the frequency and duration of subsequent spontaneous recurrent seizures as detected by chronic video-electroencephalographic (video-EEG) recordings. This finding supports a novel application for IVIg, specifically its repurposing as a disease-modifying therapy in epilepsy.

Temporal lobe epilepsy (TLE) is the most common form of focal epilepsy and is often uncontrolled by medication. Typically, an antecedent brain injury, such as febrile status epilepticus (SE), precedes the development of TLE by a period free of clinical seizures lasting several years^1^,^2^. Neurobiological changes during this period underpin epileptogenesis, the process by which the epileptic condition develops. Medications currently used to treat epilepsy primarily control the symptom of seizures, i.e. they have an anticonvulsant effect but do not consistently affect the underlying epileptogenic process. Considerable research effort is therefore focused on developing antiepileptogenic therapies to either delay or prevent the onset of the epileptic condition, or to modify the disease by reducing its progression and severity^3^.

An array of molecular and cellular changes has been reported during epileptogenesis and recent work has highlighted the role of inflammation in both experimental and human TLE^4^,^5^. Activation of microglia and astrocytes leads to the local release of pro-inflammatory mediators thought to initiate a cascade of inflammatory processes resulting in neuronal hyperexcitability and seizures^6^. In humans with focal epilepsy, both histological examination of resected tissue and positron emission tomography with ligands binding to activated microglia have provided evidence of ongoing neuroinflammation^7^,^8^,^9^. Blood-brain barrier (BBB) breakdown after the initial brain insult is well documented^10^,^11^ and is postulated to contribute to epileptogenesis by allowing entry of circulating immune cells, inflammatory molecules and albumin into the brain^12^. Breakdown of the BBB has also been observed following SE in humans and in temporal lobes removed from patients with intractable temporal lobe epilepsy^13^,^14^. We therefore explored the repurposing of an existing immunomodulatory treatment, intravenous immunoglobulin (IVIg), as an antiepileptogenic therapy.

IVIg is a sterilised and purified blood product manufactured from the pooled plasma of up to 1,000 human blood donors. It comprises mainly immunoglobulin G (IgG) (95%), the remainder being IgA with negligible concentrations of IgM^15^. It is administered intravenously and exerts an immunomodulatory effect by altering the expression and function of IgG-specific receptors (FcγR), interfering with cytokine production, and attenuating complement-mediated cell damage by binding complement activation fragments and blockade of complement receptors on mononuclear phagocytic cells^16^,^17^,^18^. In experimental models, human IVIg crosses the mouse blood-brain barrier, reaching significant concentrations in the brain^19^,^20^,^21^,^22^.

IVIg has previously been shown to be of potential benefit in epilepsies in which immunological causation is directly implicated, such as Rasmussen’s encephalitis and autoimmune limbic encephalitis^23^,^24^,^25^. In light of the potential role of inflammation and immunity in the development of epilepsy after other forms of brain injury, in the present study, we examined the effect of IVIg treatment on epileptogenesis in a mouse model of TLE arising after pilocarpine-induced SE.

## Results

### IVIg reduces microglial activation but not neuronal degeneration

**Figure 1:**
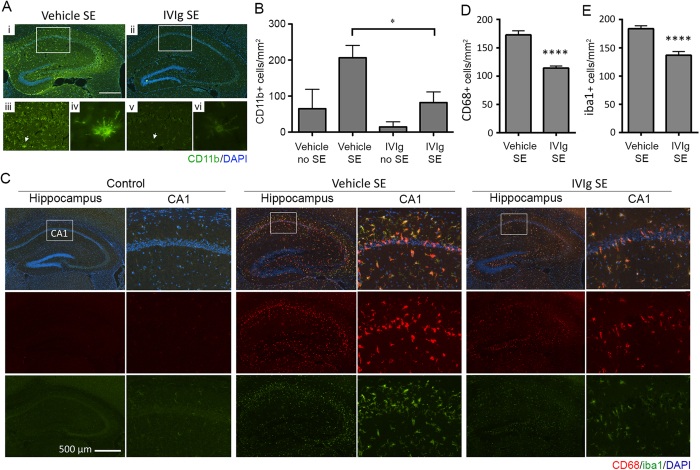
IVIg reduces microglial activation following SE. (**A**) Immunolabelling for CD11b, a microglial activation marker (*green* fluorescence), revealed intense and widespread staining throughout the hippocampus of SE animals pre-treated with vehicle (n = 7; Ai, iii), which was reduced by IVIg (n = 4; Aii, v). Cell nuclei are shown in *blue* (DAPI staining). CD11b-positive cells in vehicle-treated SE mice (Aiv, arrow) displayed the typical morphology of activated microglia, i.e. a larger cell body with short, thickened radial processes. (**B**) Quantification of CD11b-positive cell numbers in the CA1 region of the hippocampus. Note that IVIg pre-treatment significantly reduced CD11b-positive cell numbers. (**C**) Staining for CD68 (*red* fluorescence) and iba1 (*green* fluorescence) in the hippocampus of control, vehicle and IVIg post-treated SE animals. Cell nuclei are again shown in *blue*. Note the significant increase in microglial activation (CD68) and density (Iba1) in vehicle but not IVIg post-treated SE mice. (**D**,**E**) Quantification of CD68- and iba1-positive cell numbers in the CA1 region of the hippocampus of 8 vehicle post-treated SE animals and 8 IVIg post-treated animals. Scale bar = 500 μm. Data represent mean ± standard error of the mean. *p < 0.05; ****p < 0.00005.

We first determined whether IVIg therapy had an attenuating effect on microglial activation in response to pilocarpine-induced seizures. Consistent with previous reports^26^, SE was associated with a significant upregulation of CD11b on resident microglia in the hippocampus of vehicle-treated animals (Fig. 1Ai); CD11b staining was notably reduced, however, in SE animals that were pre-treated with IVIg (2 hours prior to SE induction; Fig. 1Aii). The majority of CD11b-positive microglia in vehicle-treated SE animals displayed typical activated morphology, i.e. a more obvious cell body with shortened thicker processes (arrows in Fig. 1Aiii,iv). Quantification of CD11b-positive cell numbers confirmed that IVIg pre-treatment significantly reduced the number of activated microglia in the CA1 and CA3 regions of the hippocampus compared to vehicle treatment (*p* < 0.05, one-way ANOVA with Newman-Keuls post-hoc test; Fig. 1B). Astrocyte density and morphology in the hippocampi of SE mice were similar between IVIg- and vehicle-treated animals at this time point (p = 0.21; data not shown). Reduced microglial activation was also evident when IVIg treatment was initiated 3 hours after the induction of SE (hereafter referred to as ‘post-treatment’). Specifically, quantification of CD68^+^ and Iba1^+^ cell numbers in the CA1 region of the hippocampus following post-treatment confirmed that SE-induced changes in microglial density and activation were significantly attenuated with IVIg therapy (p < 0.0001; Student’s *t*-test; Fig. 1C–E).

Quantification of Fluoro Jade C^+^ cells revealed comparable levels of neural degeneration throughout the hippocampus in vehicle-treated SE controls and mice that were pre-treated (p > 0.5; data not shown) or post-treated with IVIg (Mean ± standard error of the mean for CAI: Vehicle 509 ± 192 cells/mm^2^ vs. IVIg 938 ± 157 cells/mm^2^, p = 0.11; CA3: Vehicle 320 ± 118 cells/mm^2^ vs. IVIg 349 ± 130 cells/mm^2^, p = 0.87; dentate gyrus: Vehicle 496 ± 187 vs. IVIg: 239 ± 82 cells/mm^2^, p = 0.23; 8 mice/group).

### IVIg reduces BBB breakdown and tissue C3 levels in SE mice

**Figure 2:**
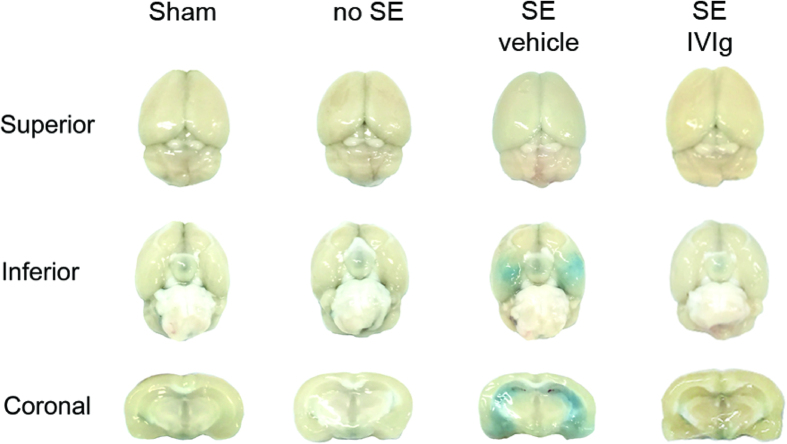
IVIg blocks SE-induced BBB breakdown. Representative images from Evans Blue dye experiments showing superior and inferior (basal) views of the brain (*top* and *middle* rows) as well as coronal sections (*bottom* row) for sham and ‘no SE’ controls, or SE mice that were post-treated with either vehicle or IVIg. Note that the prominent SE-induced breakdown of the blood-brain barrier (BBB), as evident from the extravasation of Evans Blue dye into the hippocampus, amygdala and piriform cortex, is attenuated by IVIg treatment.

BBB breakdown as a result of SE was clearly evident from Evans Blue dye extravasation experiments (Fig. 2). Sequential coronal brain sections (500 μm) of SE mice treated with vehicle solution typically showed extensive Evans Blue dye extravasation in the hippocampus, amygdala and piriform cortex. No such leakage was observed in sham-operated controls (n = 3), or in mice that did not develop SE (n = 5). Importantly, IVIg post-treatment significantly attenuated Evans Blue dye extravasation. Specifically, the proportion of ratings of 0 (no staining), 1 (mild staining) and 2 (severe staining) were 27%, 24% and 48%, respectively, in SE animals treated with vehicle control (n = 11) compared to 44%, 33% and 22% in IVIg post-treated animals (n = 9). Statistical analysis confirmed that IVIg treatment indeed ameliorated BBB breakdown, as evident from a significant reduction in the proportion of animals with severe Evans Blue staining compared to vehicle-treated SE controls (p < 0.05; Fisher-Boschloo test).

**Figure 3:**
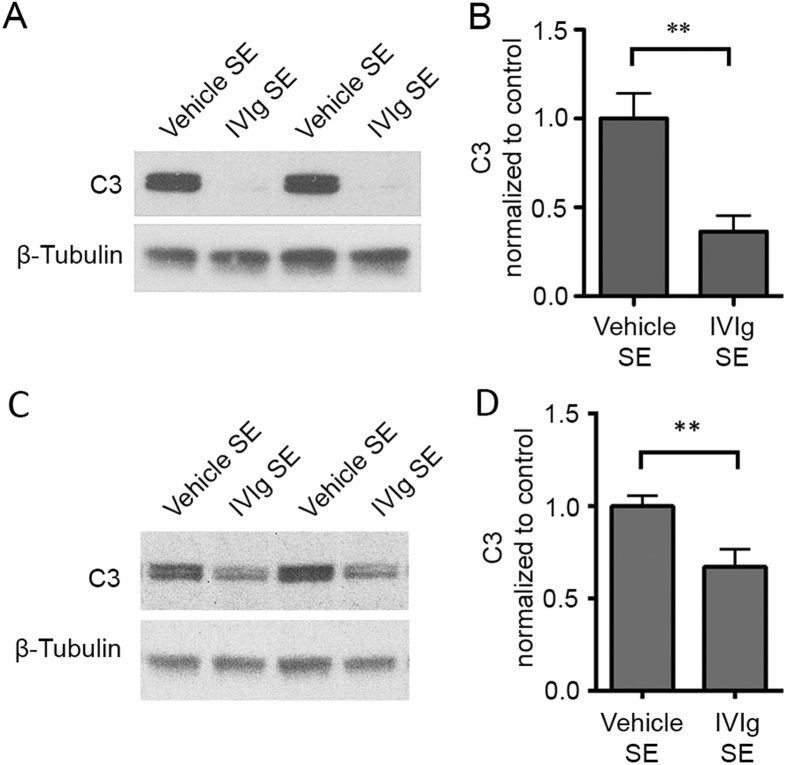
IVIg reduces C3 levels in hippocampus. Western blot analysis of C3 levels in the hippocampus of SE mice. Note that both pre-treatment (**A**,**B**) and post-treatment (**C**,**D**) of SE mice with IVIg led to a significant reduction in tissue C3 compared to that observed in vehicle-treated SE controls. Pre-treatment: 5 vehicle-treated SE animals and 4 IVIg-treated SE animals; Post-treatment: 9 vehicle-treated SE animals and 10 IVIg-treated SE animals. Data represent mean ± standard error of the mean. **p < 0.01.

In addition to reducing BBB breakdown, IVIg also ameliorated SE-associated increases in intraparenchymal levels of complement component 3 (C3), following either pre- (Fig. 3A,B) or post-treatment (Fig. 3C,D).

### IVIg post-treatment reduces the frequency of spontaneous seizures

**Figure 4:**
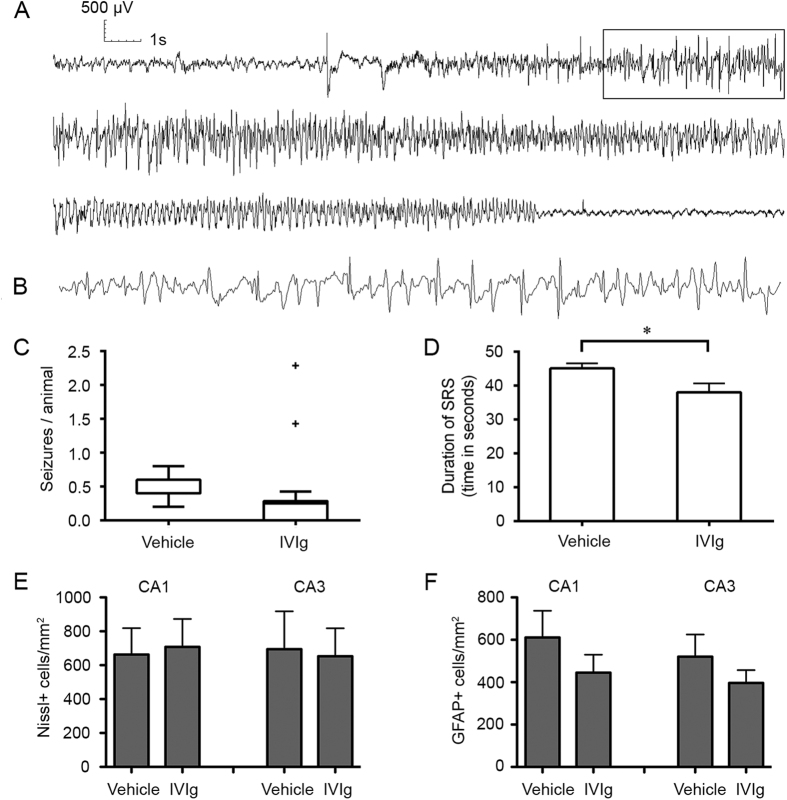
IVIg post-treatment reduces the frequency and duration of spontaneous seizures. (**A**) Typical example of a spontaneous recurrent seizure as detected on EEG recording from skull electrodes. (**B**) Higher temporal resolution image of the boxed area in (A). (**C**) Tukey box-plot of number of seizures/animal in each day in vehicle-treated (*n = 5*) and IVIg-treated (*n = 7*) mice. There was significant difference between groups [Median (interquartile range) in IVIg-treated animals: 0.25 (0–0.28) vs. vehicle-treated animals: 0.4 (0.4–0.6); p < 0.001 Mann-Whitney test, Fig. 4C]. (**D**) IVIg post-treatment significantly reduced mean seizure duration compared to that in vehicle-treated SE controls. (**E**,**F**) Post-mortem, quantitative cell counts. No significant difference in the number of Nissl (**F**) and GFAP (**G**) positive cells in the CA1 and CA3 regions of the hippocampus was observed between vehicle and IVIg-treated groups. Data represent mean ± standard error of the mean. *p < 0.05.

We next analysed IVIg’s effectiveness in counteracting epileptogenesis following the initiating insult by quantifying the frequency and duration of spontaneous seizures on EEG (Fig. 4A,B). All seizures recorded with video and EEG were electroclinical, with electrographic seizures being accompanied by behavioural manifestations, and all were secondarily generalized according to the Racine scale. All 5 vehicle-treated animals and 5 out of 7 IVIg-treated animals had spontaneous seizures during the period of video-EEG recording (p > 0.05; Fisher-Boschloo test). Seizure clusters (>6 seizures/day) occurred in two IVIg-treated animals. The number of seizures per animal was calculated for each day of monitoring and was significantly different between groups [Median (interquartile range) in IVIg-treated animals: 0.25 (0–0.28) vs. vehicle-treated animals: 0.4 (0.4–0.6); p < 0.001 Mann-Whitney test, Fig. 4C]. The proportion of days of video-EEG monitoring in which seizures occurred was also significantly lower in IVIg-treated animals (17%) than in vehicle-treated animals (42%; p < 0.001 Fisher-Boschloo test). Mean seizure duration was significantly longer in vehicle-treated (mean ± standard error of the mean: 45.1 ± 1.4 s) than in IVIg-treated animals (38.0 ± 2.6 s; two-tailed, unpaired Student’s *t*-test, p = 0.01; Fig. 4D). These differences between vehicle and IVIg-treated animals cannot be explained by differences in the severity of the initiating seizure induced by pilocarpine as Racine scale scores were similar between experimental groups (p ≥ 0.55; Table 1). Post-mortem quantification of Nissl^+^ (Fig. 4E) and GFAP^+^ (Fig. 4F) cells in the hippocampal CA1 and CA3 subfields revealed no significant differences in cell survival and astrocyte density at the study endpoint between groups post-treated with vehicle and IVIg.

## Discussion

TLE is common, affecting approximately 10% of people with epilepsy in community-based epidemiological studies^27^,^28^. A significant proportion of patients with TLE and hippocampal sclerosis are refractory to medication^29^. Hence, an effective anti-epileptogenic or disease-modifying therapy that can be administered after the initial insult is likely to have a significant impact^30^. In this study, we targeted inflammation, which is thought be an important causal mechanism in epileptogenesis, by repurposing an immunotherapeutic agent that is already in clinical use, namely IVIg.

The effect of IVIg on epileptogenesis was evaluated in the mouse pilocarpine SE model of TLE. This model has the important advantages of short latency to the development of spontaneous recurrent seizures and a high yield of epileptic animals, thereby facilitating the investigation of antiepileptogenic mechanisms and therapies^30^,^31^. Spontaneous seizures were monitored following a washout phase after termination of IVIg treatment. In doing so, we avoided any potential anticonvulsant effects of IVIg as a confounding factor in the interpretation of outcomes. IVIg did not prevent the development of epilepsy; the proportion of animals with spontaneous seizures during the period of recording was not significantly different between IVIg- and vehicle-treated groups. However, IVIg post-treatment reduced the frequency and duration of spontaneous seizures suggesting that it has a disease-modifying effect. The findings from this proof-of-concept study thus support the possibility of repurposing IVIg as a disease-modifying therapy. Extensive clinical experience has shown that IVIg therapy is safe and adverse effects associated with its administration are mostly mild and transient; there was also no indication of adverse effects following its administration in mice as part of the current study.

The mechanisms underlying the effects of IVIg treatment are not yet fully understood. However, both pre- and post-treatment with IVIg effectively blocked pathophysiological changes associated with inflammation such as microglial activation and BBB damage, both of which are thought to contribute to epileptogenesis^6^; IVIg significantly also reduced tissue C3 levels. We previously demonstrated that C3 levels in the hippocampus are elevated early after SE (48 hours to 7 days post-SE), and that C3 levels also strongly and positively correlate with the severity of the epilepsy that subsequently develops^32^. The present findings therefore strongly suggest that IVIg might alleviate epileptogenesis through modulation of the complement cascade. This is consistent with a proposed mechanism for the effect of IVIg in experimental stroke^21^,^33^ and spinal cord injury^34^ although causality remains to be proven. Hippocampal sclerosis, a common pathological change in TLE patients, is characterized by selective loss of neurons, pathological proliferation of interneuron networks and a severe glial reaction^35^. Although hippocampal damage has long been postulated to be critically involved in the development of TLE, it has not been possible to dissociate neuronal loss from gliosis with regard to disease development^36^,^37^. In our study, an effect of IVIg treatment on neuronal survival was not observed, suggesting that it modifies inflammation and the development of epilepsy through mechanisms independent of a neuroprotective effect.

Further studies are now required to inform the design of future preclinical trials involving larger animal numbers and more prolonged monitoring. Although SE is an uncommon cause of epileptogenesis in adults^30^, the preponderance of data support a causal relationship between prolonged seizures early in life, especially febrile SE, and TLE^2^. Our findings suggest that it may be possible to prevent or modify the development of TLE following early-life SE with IVIg administered post-insult. Further experiments are also required to examine IVIg’s effects on epileptogenesis due to other causes such as traumatic brain injury, and to prove causality between the observed effects of IVIg treatment on inflammation (i.e. microglial activation, C3 expression and BBB breakdown) and the subsequent reduction in spontaneous recurrent seizures. Future experiments with modified immunoglobulin may allow the responsible parts of the molecule (e.g. the variable antigen-binding portion, the F(ab′)2 fragment, and the crystallisable domain, the Fc fragment) to be elucidated. The most effective IVIg post-treatment regime also requires further elucidation to maximise cost-effectiveness and to minimise the risk of adverse effects.

In summary, we have demonstrated a disease-modifying effect of IVIg post-treatment in an experimental model of TLE. This approach of repurposing an established immunotherapeutic agent has the potential for rapid translation to clinical trials.

## Methods

Experimental procedures were approved by The University of Queensland Animal Ethics Committee (AEC Approval Number: CAI/088/13/NHMRC) and conducted in accordance with the relevant policies and guidelines of the Australian National Health and Medical Research Council. Animals were housed individually under controlled laboratory conditions (12 hours light/12 hours dark cycle, with lights on at 07:00 a.m., temperature 22 ± 1 °C, air humidity 50–60%) and had *ad libitum* access to food and water.

### Pilocarpine Model and Behavioural Analysis

**Figure 5:**
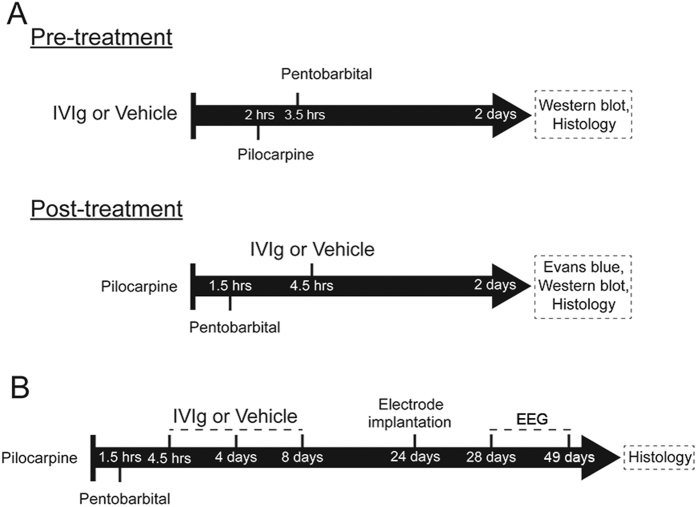
Study design. Schematic overview of the experimental design and timing (i.e. pre- or post-treatment) of IVIg administration for acute (**A**) and chronic (**B**) studies.

Adult outbred CD1 mice were injected with pilocarpine s.c. (330 mg/kg, Sigma-Aldrich, St Louis, USA) to induce SE, 30 minutes after the injection of methylscopolamine i.p. (2.5 mg/kg, Sigma-Aldrich). Animals were observed in real-time for behavioural signs of seizure activity for 1.5 hours before the injection of pentobarbital (30 mg/kg, Troy Laboratories, Australia) (Fig. 5A,B). Seizure severity was scored according to the modified Racine scale: normal activity (stage 0); rigid posture and/or immobility (stage 1), stiffened, extended, arched (Straub) tail (stage 2); unilateral forelimb clonus or head bobbing (stage 3); whole body continuous clonic seizures with retained posture (stage 3.5); bilateral forelimb clonus and rearing (stage 4); rearing and falling (stage 5); and tonic-clonic seizures with loss of posture control or jumping (stage 6). Stage 3.5–6 seizures were considered generalized. SE was defined as a minimum of three stage 4–6 seizures or at least 30 min of continuous seizures (at least stage 3.5) with one or more stage 5 or 6 seizures^32^,^38^. The latency to development of the first seizure was also timed and recorded.

### IVIg Administration

We used a dose of 2 g/kg IVIg as this dose is used clinically and was also previously shown to be neuroprotective in animal models of stroke^21^ and spinal cord injury^34^. A summary overview of the experimental design is shown in Fig. 5. For the pre-treatment experiments, animals were randomly allocated to groups treated with IVIg (Baxter Healthcare, Brisbane, Australia, 2 g/kg), or the same volume of vehicle solution (5 mg/ml glycine, used as a stabilizing agent for IVIg) by femoral vein injection 2 hours before pilocarpine administration (*pre-treatment*; Fig. 5A). The mortality in vehicle-treated and IVIg pre-treated animals was 20% (4 of 20 animals) and 5% (1 of 20 animals) respectively. Severity (p = 0.22) and latency (p = 0.70) of the seizures were not significantly different between animals developing SE in IVIg- and vehicle-treated groups. Animals were sacrificed for immunohistochemistry and western blotting 2 days after pilocarpine SE.

For the post-treatment experiments, SE animals were randomly allocated to receive IVIg or vehicle solution 3 hours after pilocarpine administration by an investigator who was blinded to data on the severity of the pilocarpine-induced seizures (*post-treatment*; Fig. 5A). Western blotting, immunostaining, and Evans Blue studies were conducted 2 days post-SE in separate groups of animals (Fig. 5A).

To examine a putative antiepileptogenic effect of IVIg in longer term studies, SE animals were randomly allocated to two groups receiving either vehicle or IVIg 3 hours after the cessation of the motor seizures by pentobarbital (Fig. 5B). As the serum concentration of human IgG following intravenous administration in mice reaches 50% of the initial concentration after 3–4 days and is undetectable after 20 days^39^, IVIg was re-administered at 4 and 8 days to ensure that circulating human IgG levels were maintained for the first 10 days after SE. EEG recording was conducted from 20 days after the last IVIg injection onward (washout phase), which eliminates anticonvulsant actions of IVIg as a potential confounding factor. Animals were sacrificed after EEG recording and histopathological examination of their brains performed. The total number of animals used in this study and the initial seizure scores of SE groups post-treated with either vehicle or IVIg are detailed in Table 1. In both pre- and post-treatment experiments, veterinary welfare monitoring by researchers and animal house staff did not identify any adverse effects related to IVIg administration.

### Immunofluorescent Staining

Brain sections were blocked with 10% goat serum in PBS containing 0.2% Triton X-100 and then immunostained with microglial marker mouse anti-CD11b (1:200, Abcam, Cambridge, UK), rabbit anti-Iba1 (1:400, Wako Chemical), rat anti-CD68 (1:200, AbD Serotec) and astrocyte marker mouse anti-GFAP (1:200, Merck Millipore) in blocking solution at 4 °C overnight. Omission of the primary antibody incubation step and/or isotype-matched control antibodies was used to ascertain staining specificity. Brain sections were then incubated at room temperature for 1 hour with AlexaFluor goat anti-mouse IgG-488, goat anti-rabbit IgG-488 and goat anti-rat IgG-555 (Molecular Probes, Life Technologies, USA) at a dilution of 1:200. Brain sections were counterstained with DAPI (4′,6-diamidino-2-phenylindole, 1:2000, Sigma) and mounted using Prolong Gold (Molecular Probes).

### Evans Blue Staining

Evans Blue was administered by i.p. injection (2% in saline solution, 4 ml/kg of body weight) 24 hours post-SE. Mice were transcardially perfused with 50 ml of ice-cold PBS (Invitrogen) 48 hours post-SE, after which the brains were dissected and photographed. Three investigators, blinded to treatment, rated the level of brain tissue staining as no staining (score 0), mild staining (mild dye leakage, score 1), and severe staining (marked dye extravasation, especially in hippocampus, amygdala and piriform cortex, score 2).

### Western Blotting

The presence of complement component C3 (C3) in hippocampal tissue homogenates (40 μg/μl protein samples) was assessed and quantified using Western blot as previously described^21^. Western blots were probed using rat anti-C3 (1:200, Hycult biotech, Netherlands) antibody. Rabbit anti-β-tubulin (1:2000, IMGENEX, Bhubaneswar, India) was used to control for protein loading.

### Fluoro Jade C Staining

Brain slides (14 μm cryostat brain sections) were immersed for 3 min in 100% ethanol, 1 min in 70% ethanol, 1 min in distilled water, and then transferred to a solution containing 0.0004% Fluoro-Jade C (FJC, Merck Millipore, Germany) and 0.1% acetic acid for 30 min. After three washes in distilled water, the slides were dried overnight. The slides were then immersed in xylene 3 times for 2 min each and mounted with DPX mounting medium (Sigma-Aldrich).

### Nissl Staining

Brain sections were immersed in 100% ethanol 3 times for 3 minutes each, followed by 100% xylene twice for 15 minutes and then 100% ethanol for 10 minutes. Next, sections were washed briefly in water, stained in 0.1% Cresyl Violet for 4 minutes, washed again in water to remove excess stain and dehydrated through 2 changes of absolute ethanol for 3 minutes. Sections were then cleared in xylene and mounted with DPX.

### Histological Image Acquisition and Analysis

Images were obtained using a Nikon Inverted Fluorescent Microscope (Nikon, Tokyo, Japan). The number of CD11b-, Iba-1-, CD68-, GFAP-, and Nissl-positive cells were quantified in the CA1 and CA3 subregions of the dorsal hippocampus (−1.46 and −1.70 mm from bregma) using the NIS elements AR imaging software (Nikon, Tokyo, Japan).

### EEG Recording and Analysis

Electrode implantation was performed at 24 days post-SE and continuous video-EEG recording commenced 4 days later, i.e. at 28 days (Fig. 5B) as previously reported^32^. Four stainless steel epidural screw electrodes (1 mm diameter, Microbiotech/SE AB, Sweden) were inserted in the skull over the left and right parietal cortex and over the cerebellum bilaterally and served as two recording electrodes and ground and indifferent electrodes respectively. We analysed data recorded continuously over a 3 week period in 7 IVIg post-treatment SE animals and 5 vehicle-treated SE controls. Detection of electrographic seizures was performed by investigators who were blinded to the experimental condition/treatment. Screening of EEG recordings for possible electrographic seizures was performed by QT with subsequent confirmation by DR and MC. An electrographic seizure was defined as a high amplitude (>2 × baseline) rhythmic discharge or spike and wave pattern with a clear onset, offset, and temporal evolution in frequency and/or morphology, lasting >10 s (Fig. 4A to B)^32^. Seizures were confirmed and behavioural severity of seizures was assessed on video according to the modified Racine scale^32^. Three animals from the IVIg group had video-EEG recording until day 10 and only video recording thereafter because of separation of their electrode headsets. All available data were examined to detect seizures in all animals. The number of seizures per animal was calculated for each day of recording and seizure duration was analysed on available EEG recordings of seizures.

### Statistical Analysis

The Fisher-Boschloo exact unconditional test^40^,^41^ was used to analyse contingency tables. This test was used to determine the statistical difference between vehicle and IVIg treated groups in the rating of severity of Evans Blue staining and in the proportion of days in which seizures occurred. Statistical differences between experimental conditions for continuous and count data (Western blotting, histological cell counts, video-EEG data on number of seizures per day and seizure duration) were determined using two-tailed, unpaired Student’s *t*-tests, one-way ANOVA with the post-hoc Newman-Keuls Multiple Comparison test or the Mann-Whitney test as appropriate (GraphPad Prism V5). For all analyses, statistical significance is reported at a threshold of *p* < 0.05.

## Additional Information

**How to cite this article**: Chen, M. *et al*. Disease-modifying effect of intravenous immunoglobulin in an experimental model of epilepsy. *Sci. Rep.* **7**, 40528; doi: 10.1038/srep40528 (2017).

**Publisher's note:** Springer Nature remains neutral with regard to jurisdictional claims in published maps and institutional affiliations.

## Figures and Tables

**Table 1:** Mice used in pre- and post-treatment experiments.

Numbers of adult CD1 mice							
Pre-treatment			Post-treatment				
*Groups*	*IHC*	*WB*	*Groups*	*IHC/FJC*	*WB*	*EB*	*EEG*
Vehicle	12	8	**Pilocarpine**	24	45	39	29
IVIg	13	7	**SE mice**	16	19	20	18
Vehicle SE	7	5	*Vehicle*	8	9	11	5
				(21.8 ± 1.6)	(21.6 ± 2.2)	(20.6 ± 1.8)	(21.5 ± 2.8)
IVIg SE	4	4	*IVIg*	8	10	9	7
				(21.0 ± 2.5)	(20.7 ± 1.3)	(21.9 ± 3.4)	(24.2 ± 3.1)

In pre-treatment experiments, mice were randomly allocated to receive vehicle or IVIg before administration of pilocarpine. Animals developing SE were used for immunohistochemistry (IHC), Fluoro Jade C staining (FJC) and western blotting (WB) studies, as shown in the bottom two rows.

In post-treatment experiments, mice developing SE were randomly allocated to receive vehicle or IVIg. The number of animals receiving pilocarpine (Post-treatment, Row 1), the number developing SE (Row 2) are shown. The number of animals receiving vehicle or IVIg used for IHC, WB, Evans blue staining (EB) and electroencephalography (EEG) are shown in Rows 3 and 4. The numbers in brackets are the seizure scores of each group (modified Racine scale, mean ± standard error of the mean, no significant difference between SE vehicle and SE IVIg groups, unpaired *t*-test). Fewer animals underwent EEG recording than were randomised due to the death of 3 vehicle-treated and 3 IVIg-treated animals before EEG recording.
